# Supplementary material for: Long-Term Results with Everolimus in Advanced Hormone Receptor Positive Breast Cancer in a Multicenter National Real-World Observational Study
Source: Cancers (Basel). 2023 Feb 13;15(4):1191. doi: 10.3390/cancers15041191 (PMC9954606; doi:10.3390/cancers15041191)
Supplement: Supplementary file 1 [file cancers-15-01191-s001.zip › cancers-2121345-supplementary.pdf]

cancers

MDPI

Supplementary

Long-Term Results with Everolimus in Advanced Hormone Receptor Positive Breast Cancer in a Multicenter National Real-World Observational Study

A

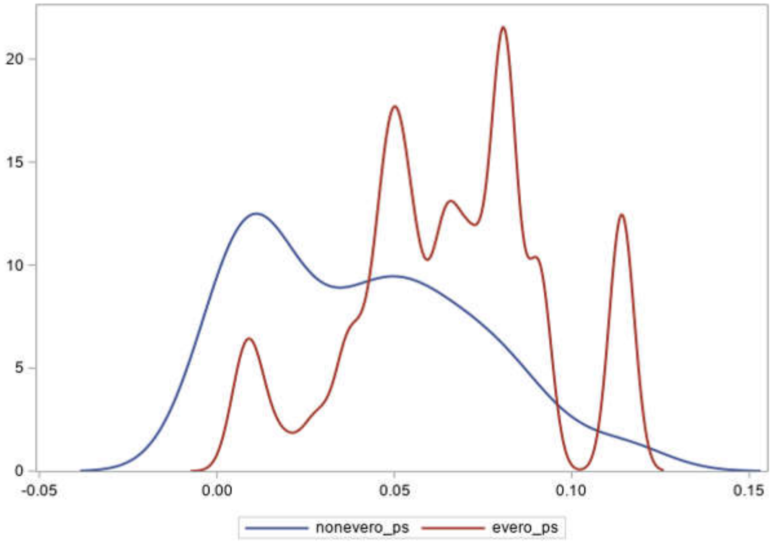

B

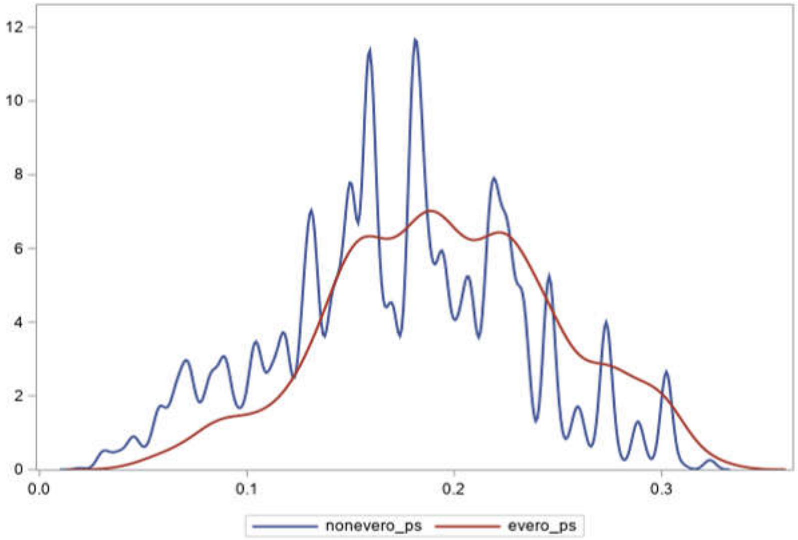

C

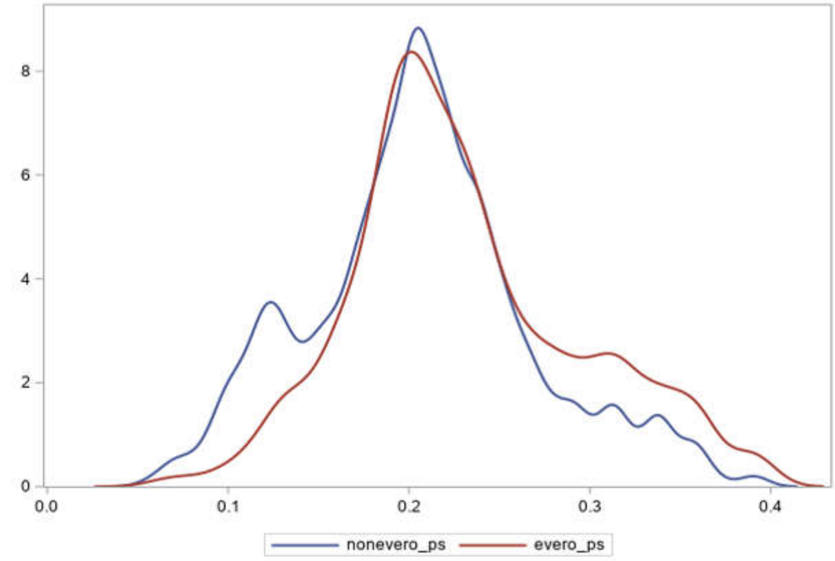

Figure S1. Harrell's C index kernel density plotting. (**A**). Patients in L1. (**B**) Patients in line 2. (**C**) Patients in line 3 and beyond. OS: overall survival. Nonevero_ps: propensity score distribution of patients who did not receive everolimus (blue curve). Evero_ps: propensity score distribution of patients exposed to everolimus (red curve).

A

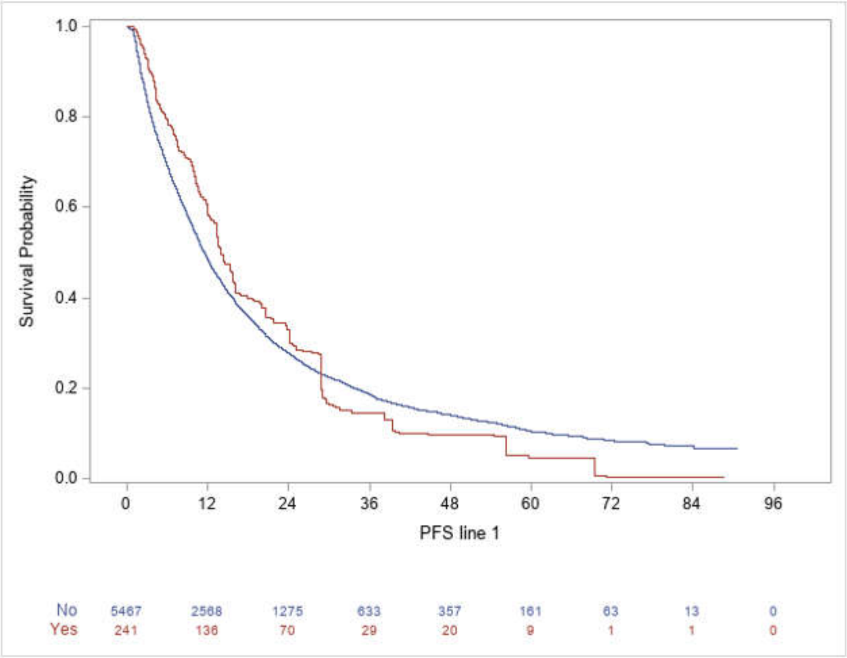

B

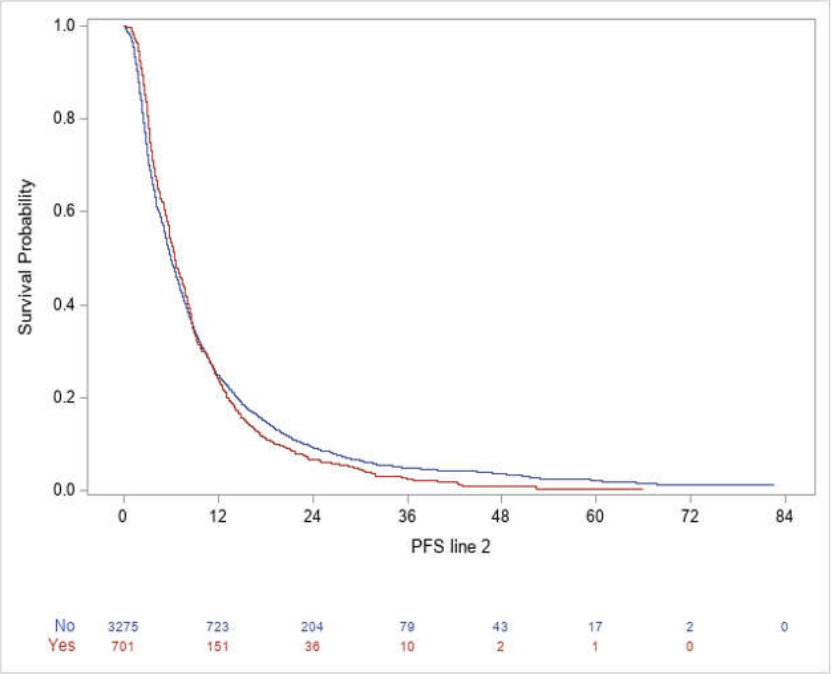

C

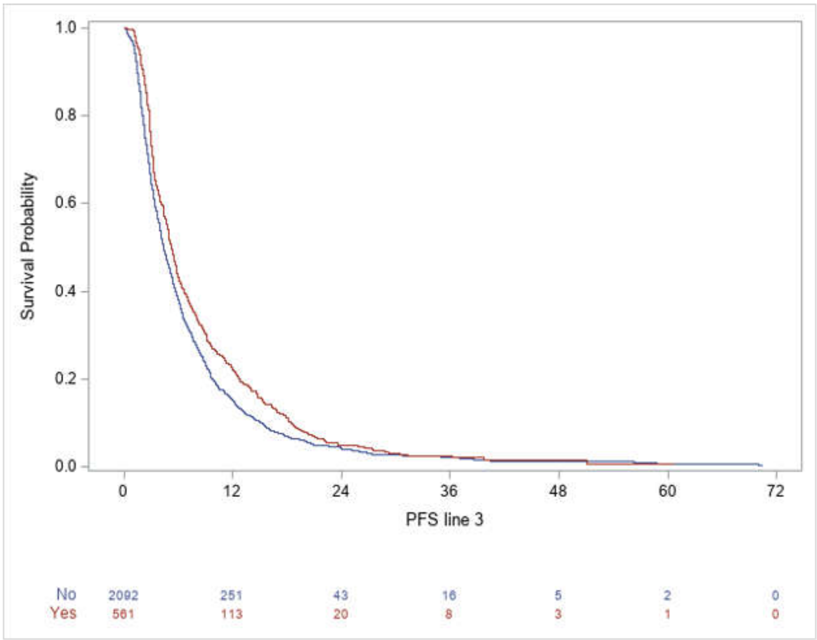

Figure S2. Progression-free survival as per inverse probability of treatment weighting analysis. (**A**) Patients in L1. (**B**) Patients in line 2. (**C**) Patients in line 3 and beyond. PFS: progression-free survival. No: patients who did not receive everolimus (blue curves). Yes: patients exposed to everolimus (red curves).

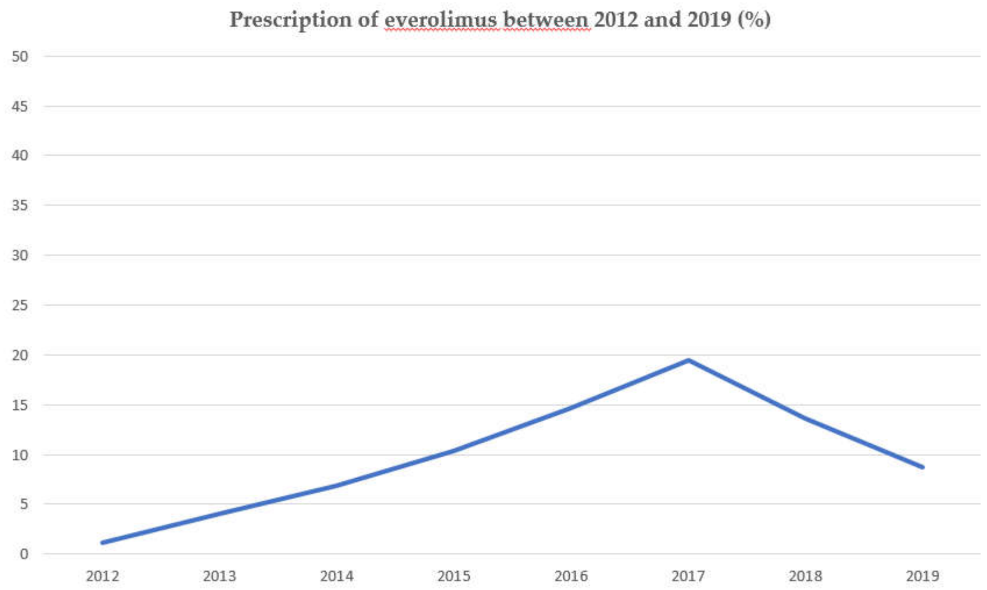

**Year**	**2012**	**2013**	**2014**	**2015**	**2016**	**2017**	**2018**	**2019**
**N**	19	116	260	456	667	788	349	99
**%**	1.16	4.0	6.8	10.4	14.7	19.5	13.6	8.7

Figure S3. Proportion of patients receiving everolimus in the advanced setting between 2012 and 2019.
